# Sellar Atypical Teratoid/Rhabdoid Tumors (AT/RT): A Systematic Review and Case Illustration

**DOI:** 10.7759/cureus.26838

**Published:** 2022-07-14

**Authors:** Kimberly Major, Lekhaj C Daggubati, Christine Mau, Brad Zacharia, Michael Glantz, Cunfeng Pu

**Affiliations:** 1 Neurosurgery, Penn State Health Milton S. Hershey Medical Center, Hershey, USA; 2 Pathology, Penn State Health Milton S. Hershey Medical Center, Hershey, USA

**Keywords:** adult, sellar atypical teratoid/rhabdoid tumor, at/rt, sellar at/rt, atypical teratoid rhabdoid tumors

## Abstract

Introduction: Atypical Teratoid/Rhabdoid tumors are rare, highly malignant tumors in adults, with a median survival of 20 months. We report a case of a sellar atypical teratoid/rhabdoid tumor in a 70-year-old female treated with intraventricular chemotherapy, followed by a systematic review of the current management of sellar AT/RTs.

Methods: A comprehensive systematic literature search was conducted on Web of Science, Scopus, and PubMed Central using the key terms “sellar” and “atypical teratoid/rhabdoid tumors”, following Preferred Reporting Items for Systematic Reviews and Meta-Analyses (PRISMA) guidelines. Data, including patient demographics, histology, treatments, and overall survival were extracted and analyzed. Kaplan-Meier survival curves and log-rank analysis were used to compare survival outcomes between different treatment regimens.

Results: Our literature search disclosed 123 publications. After prespecified exclusions, 41 patients with sellar AT/RT from 30 manuscripts were identified, and 38 were included in the final analysis. Including our patient, the median age was 44 (range: 20-70) with a substantial female predominance (94.7%). Collectively, patients who received combined chemoradiation therapy had a significantly increased overall survival compared to those who received single modality or no adjuvant therapies (median OS 27 vs. 1.25 months; *p*=0.0052).

Conclusion: Atypical teratoid/rhabdoid tumor in the sellar region carries a poor prognosis. Adjuvant chemotherapy and radiation therapy were associated with significantly increased overall survival. Early consideration of neuro-oncology and radiation-oncology referral and management is likely beneficial in this patient population. Intrathecal chemotherapy is a treatment modality that requires further exploration given the limited options and current dismal prognosis of adult sellar AT/RT.

## Introduction and background

Atypical Teratoid/Rhabdoid Tumors (AT/RT) are rare, highly malignant tumors of the central nervous system, primarily occurring in children younger than three years old. These tumors are characterized by deletions in the SMARCB1 (INI1) or SMARCA4 (BRG1) tumor suppressor genes on chromosome 22q11.2, resulting in the loss of INI1 or BRG1 expression [1,2]. In the pediatric population, the prognosis is grim, with median survival between six and 18 months [1-5]. Treatment includes a multimodal approach involving maximal safe resection, chemotherapy, and radiation therapy, which has been shown to improve survival [6-11].

Adult cases of AT/RT are rare, with fewer than 50 cases previously reported in the literature. Median survival in adults ranges between 10 and 48 months, with increased survival achievable using treatment paradigms utilized in children [12-15]. Sellar AT/RTs, while uncommon in children, is the most frequent location in adults. Despite the predominance of sellar AT/RT in the adult population, very little has been reported specifically on this entity. To this end, we report a case of sellar AT/RT followed by the first systematic review on the management of sellar AT/RTs.

## Review

Materials & methods

A comprehensive systematic literature search was conducted on Web of Science, Scopus, and PubMed Central using the key terms “sellar” and “atypical teratoid/rhabdoid tumors”, following Preferred Reporting Items for Systematic Reviews and Meta-Analyses (PRISMA) guidelines. Reference lists of all relevant publications were reviewed. Duplicate publications were removed. The title and abstracts were examined to identify potential eligible articles before full-text evaluation. Case reports and series of adult patients (>18 years old) diagnosed with sellar AT/RT in which follow-up data were complete were included in the quantitative analysis. Full-text articles were independently reviewed by two of the authors. Patients’ age, sex, presence of INI-1 alterations, extent of resection, chemotherapy regimens, and radiation therapy regimens were collected. SAS version 9.4 (SAS Institute, Cary, NC) was used to analyze the data, including descriptive statistics and Kaplan-Meier survival curves. The log-rank test was used to compare overall survival (OS) between the treatment groups of interest. This research was conducted under protocol STUDY00005691, Penn State Hershey Brain, Skull Base, Spine and Peripheral Nervous System Tumor Registry, approved by the Penn State Institutional Review Board.

Systematic review

**Figure 1:**
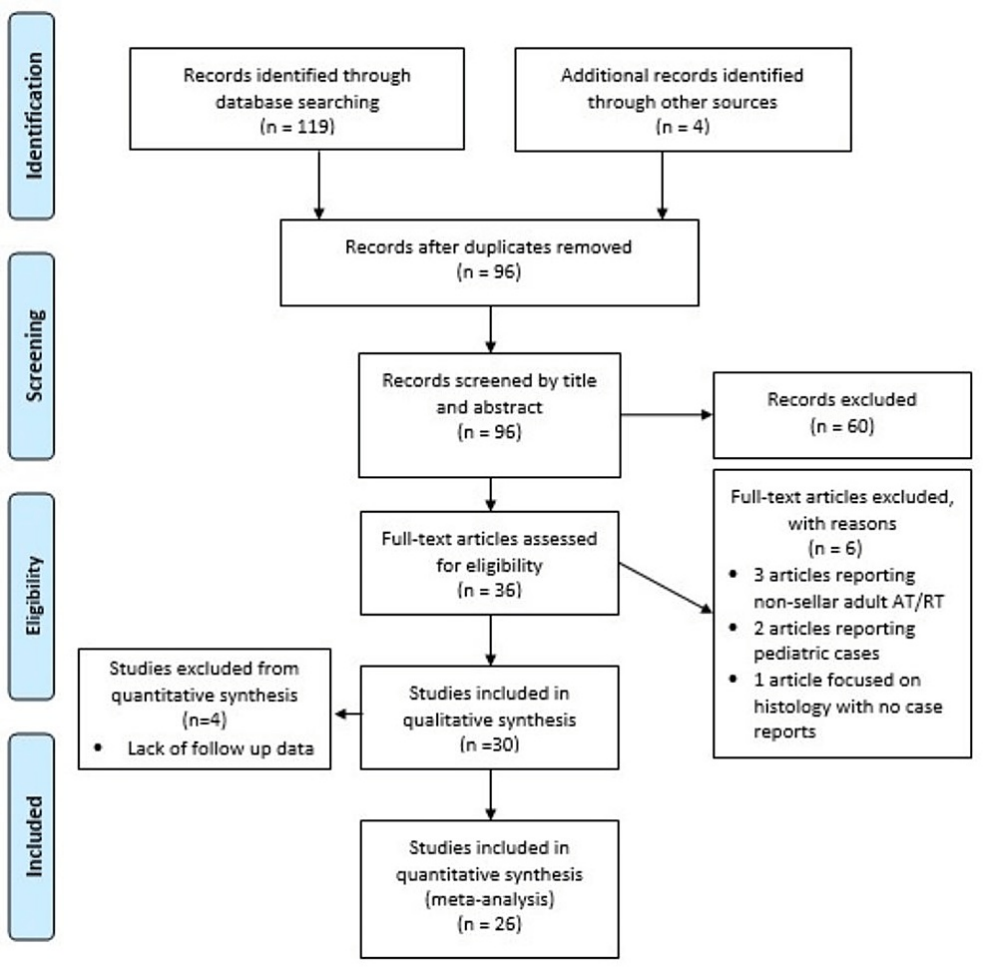
PRISMA flow diagram AT/RT: Atypical Teratoid/Rhabdoid Tumor

The literature search resulted in 123 published works (Figure 1).

After duplications were removed, 96 studies were screened, with additional 60 studies excluded. The studies excluded during the screening process included articles not specific to sellar AT/RT and those with full length articles not available. Following the review of full-length articles, six more studies were excluded that focused on pediatric AT/RT, histology of AT/RT, genetics of AT/RT, or did not report AT/RT case information. After exclusions, 30 publications were deemed relevant, resulting in 41 individual cases (Table 1) [13-42]. Four cases were excluded from quantitative analysis due to incomplete follow-up information for a total of 38 cases, including the case reported in this manuscript.

**Table 1:** Qualitative review of the literature, including patient demographics, MRI findings, expression of INI1, reported treatment regimen, and overall survival. GTR: gross total resection, STR: subtotal resection, NR: not reported, MRI: magnetic resonance imaging, HA: headache, AMS: altered mental status, SAH: subarachnoid hemorrhage, IVH: intraventricular hemorrhage, CN: cranial nerve; * not included in quantitative review

Published study	Age	Sex	Loss of INI1	Duration of symptoms	MRI Image Findings	Treatment	Follow-up (months)	Evidence of dissemination
Kuge et al. 2000 [13]	32	F	NR	NR	Contrast enhancing sellar lesion	GTR; cisplatin, etoposide, interferon; craniospinal and focal radiotherapy	28 (deceased)	Yes
Raisanen et al. 2005 [14]	20	F	Yes	NR	2.0 × 1.9 cm, partially cystic, heterogeneously enhancing sellar lesion	Resection, unspecified extent; unspecified chemotherapy and radiation therapy	28 (alive)	No
Raisanen et al. 2005 [14]	31	F	Yes	NR	1.6-cm enhancing sellar/suprasellar lesion	Resection, unspecified extent; unspecified radiation therapy	9 (deceased)	Yes
Arita et al. 2008 [15]	56	F	Yes	HA and diplopia for 2 months	Heterogeneously enhancing sellar lesion; right cavernous sinus invasion	STR, stereotactic radiation (total=51 Gy)	23 (deceased)	Yes
Las Heras et al. 2010* [16]	46	F	Yes	NR	NR	Resection, unspecified extent	NR*	NR*
Schneiderhan et al. 2011 [17]	61	F	Yes	NR	Heterogeneously enhancing sellar/suprasellar mass with parasellar expansion; edema of the adjacent brain parenchyma and bilateral optic tracts	STR x2	3 (deceased)	No
Schneiderhan et al. 2011 [17]	57	F	Yes	NR	Heterogeneously enhancing sellar lesion with right-sided parasellar expansion	GTR; doxorubicin and cisplatin; unspecified radiation	6 (alive)	No
Chou et al. 2013 [18]	43	F	Yes	HA and diplopia for 10 days	Isointense/hypointense sellar lesion with heterogeneous enhancement; invasion into the left cavernous sinus	STR, radical radiotherapy, unspecified	0.5 (deceased)	Yes
Moretti et al. 2013 [19]	60	F	Yes	NR	Heterogeneously enhancing sellar lesion with extrasellar; left cavernous sinus invasion, encasing the internal carotid artery	STR; doxorubicin, vinorelbine, carboplatin and paclitaxel; stereotactic radiotherapy (total=51 Gy)	30 (deceased)	Yes
Park et al. 2014 [20]	42	F	Yes	NR	Heterogeneously enhancing solid and cystic sellar/suprasellar mass.	STR; cisplatin, doxorubicin, vincristine, etoposide, ifosfamide, cyclophosphamide; Craniospinal, proton beam, and boost radiotherapy (total=54 Gy)	27 (alive)	NR
Shitara et al. 2014 [21]	44	F	Yes	2 months visual disturbance	Heterogeneously enhancing lesion	STR x 2; ifosfamide, cisplatin and etoposide; unspecified radiotherapy	17 (deceased)	Yes
Lev et al. 2015 [22]	36	F	Yes	HA 1-month, blurry vision 6 days	3.3 × 3.2 × 2.3 cm heterogeneously enhancing sellar lesion; compression of the optic chiasm, left cavernous sinus invasion	STR x 6; temozolomide, cyclophosphamide, adriamycin, vincristine, cisplatin, etoposide; external beam radiotherapy to sellar region	29 (deceased)	No
Biswas et al. 2015 [23]	48	F	Yes	2-week visual field disturbance	Sellar lesion with “malignant characteristics”	STR; vincristine, doxorubicin, cyclophosphamide alternating with ifosfamide, carboplatin, etoposide; pituitary and craniospinal radiotherapy	2 (deceased)	Yes
Regan et al. 2015 [24]	45	F	Yes	HA, diplopia 9 days	Hypointense sellar lesion with extension into the left cavernous sinus	STR; stereotactic radiotherapy to cavernous sinus and parasellar region	6 (deceased)	No
Nobusawa et al. 2016 [25]	69	F	Yes	NR	2.8x 1.6 cm isointense sellar lesion, extension into the left cavernous sinus surrounding the internal carotid artery	STR; temozolomide; focal radiotherapy	24 (alive)	No
Almalki et al. 2016 [26]	36	F	Yes	HA 3 months, diplopia 1 month	Heterogeneously enhancing sellar/suprasellar lesion; Bilateral invasion of cavernous sinus and clivus with posterior destruction of clinoid	STR; vincristine & fractionated radiotherapy (60 Gy in 30 fractions) followed by ifosfamide, cisplatin and etoposide	37 (alive)	No
Larran-Escandon et al. 2016 [27]	43	F	Yes	HA 3 months, diplopia and ptosis 2 weeks	2.0 x 2.3 cm sellar/suprasellar lesion with subacute hemorrhage	STR	1 (deceased)	No
Elsayad et al. 2016 [28]	66	M	NR	NR	2.2 × 1.6 × 1.4 cm heterogeneously enhancing sellar lesion	STR followed by GTR; fractionated radiotherapy (59.4 Gy in 33 fractions)	48 (alive)	No
Nakata et al. 2017 [29]	31	F	Yes	NR	NR	Resection, unspecified extent; cisplatin and etoposide followed by methotrexate (intrathecal); local and posterior fossa radiotherapy	28 (deceased)	NR
Nakata et al. 2017 [29]	56	F	Yes	NR	NR	Resection, unspecified extent; stereotactic radiosurgery, craniospinal radiotherapy	23 (deceased)	Yes
Nakata et al. 2017 [29]	44	F	Yes	NR	NR	Resection, unspecified extent; ifosfamide, cisplatin, and etoposide; unspecified radiotherapy	17 (deceased)	NR
Nakata et al. 2017 [29]	26	F	Yes	NR	NR	Resection, unspecified extent; methotrexate (intrathecal) followed by ifosfamide, cisplatin, and etoposide; local and spine radiotherapy	33 (deceased)	Yes
Nakata et al. 2017 [29]	21	F	Yes	NR	NR	Resection, unspecified; ifosfamide, cisplatin, etoposide; local radiotherapy	35 (deceased)	NR
Nakata et al. 2017 [29]	69	F	Yes	NR	NR	Resection, unspecified; temozolomide; local radiotherapy	37 (alive)	NR
Dardis et al. 2017 [30]	35	M	Yes	3 months blurred vision	Mixed cystic/solid heterogeneously enhancing suprasellar and interpeduncular lesion	STR followed by GTR; fractionated craniospinal radiotherapy with localized boosts with cisplatin sensitizer (total=66 Gy in 36 fractions); high dose cyclophosphamide and vincristine followed by autologous stem cell transplant	30 (alive)	Yes
Pratt et al. 2017* [31]	47	F	Yes	NR	2.6 x 3.9 x 3.2 cm heterogeneously enhancing sellar mass; erosion of the surrounding bone and extension into bilateral cavernous sinuses. Complete encasement of left carotid artery.	Resection, unspecified extent	NR*	NR*
Johann et al. 2018 [32]	20	F	Yes	NR	NR	Resection, unspecified extent; high dose chemotherapy (ifosfamide, cisplatin, etoposide) followed by autologous stem cell rescue	120 (deceased)	NR
Nishikawa et al. 2018 [33]	42	F	Yes	Slight headache on presentation (unknown duration); severe headache, vertigo and visual disturbance after 2 months conservative therapy	1.9 x 2.0 x 0.5 cm intrasellar mass, left cavernous sinus invasion, optic chiasm compression	STR; temozolomide; stereotactic radiation x2 (total= 30 Gy); recurrence with STR 6 months after initial surgery followed by paclitaxel and conventional radiotherapy to residual	11 (deceased)	Yes
Paolini et al. 2018 [34]	31	F	Yes	NR	Heterogeneous enhancing sellar/suprasellar lesion	STR	2 (deceased)	NR
Paolini et al. 2018 [34]	36	F	Yes	NR	NR	STR; unspecified chemotherapy and radiotherapy	22 (alive)	NR
Paolini et al. 2018 [34]	46	F	Yes	NR	NR	STR	0 (deceased)	NR
Paolini et al. 2018 [34]	47	F	Yes	NR	NR	STR; 3 agent chemotherapy; fractionated radiotherapy (20 Gy in 10 fractions)	62 (alive)	NR
Paolini et al. 2018 [34]	65	F	Yes	NR	NR	STR; vincristine, cisplatin, doxorubicin and cyclophosphamide; fractionated radiotherapy with cisplatin sensitizer (54 Gy in 30 fractions)	23 (deceased)	Yes
Barresi et al. 2018 [35]	59	F	Yes	NR	2.3 x 1.2 cm heterogeneously enhancing sellar lesion; invasion in to left cavernous sinus	STR; unspecified radiotherapy	2 (deceased)	No
Su et al. 2018 [36]	37	F	Yes	2 months blurred vision	Heterogeneously enhancing 2.57 x 1.96 x 3.63 cm sellar/suprasellar lesion	STR	1 (deceased)	NR
Barsky et al. 2018* [37]	54	F	Yes	NR	1.6 x 1.1 x 2.4 cm sellar/ suprasellar lesion with edema	STR	NR* (alive)	No*
Asmaro et al. 2019 [38]	62	F	Yes	HA and diplopia several months	Heterogeneously enhancing sellar/suprasellar hemorrhagic lesion, with SAH and IVH	STR	2 (deceased)	NR
Voison et al. 2019 [39]	51	F	Yes	5 months visual disturbance	Preoperative CT: lobulated heterogeneously enhancing suprasellar cystic lesion	STR; temozolomide and focal radiotherapy followed by fractionated craniospinal radiotherapy and photo beam radiation (total radiation= 54 Gy) 1 dose of ifosfamide, carboplatin, and etoposide	9 (alive)	No
Siddiqui et al. 2019 [40]	55	F	Yes	1 week HA, blurred vision, acute AMS	Hemorrhagic sellar mass with SAH and IVH	GTR	1.5 (deceased)	NR
Lawler et al. 2019* [41]	27	F	Yes	NR	Enlarged pituitary fossa and gland with an ill-defined lesion at the floor of the pituitary fossa	Resection, unspecified extent	NR*	NR*
Bokhari et. al 2020 [42]	40	F	Yes	NR	2.9 × 1.7 × 2.3 cm sellar enhancing cystic lesion	STR, unspecified chemotherapy and radiotherapy	1 (deceased)	NR
Present report	70	F	Yes	3-month HA, 4 months right eye vision changes, acute right CNIII palsy	1.8 x 2.2 x 1.7 cm heterogeneously enhancing sellar/suprasellar mass with invasion into the right cavernous sinus encasing the right internal carotid artery	STR x 2; intravenous carboplatin and etoposide x 1 infusion; alternating intrathecal etoposide plus topotecan and intrathecal methotrexate, and thiotepa (3 cycles); fractionated external beam focal radiotherapy (30 Gy in 10 fractions)	5.5 (deceased)	Yes

Median age was 44 years (range 20-70) with a female to male ratio of 18:1 (Table 2).

**Table 2:** Patient demographics and summative descriptive data SD: standard deviation, IQR: interquartile range

Patient Demographics	
N	38
Mean age (SD)	45.6 (14.2)
Median age (IQR)	44 (36-56.8)
Female sex no. (%)	36 (94.7)
Treatment	
Extent of resection no. (%)	
Gross total resection	5 (13)
Subtotal resection	24 (63)
Resection, unknown extent	9 (24)
Adjuvant therapies no. (%)	
Chemoradiation	23 (60.5)
Chemotherapy only	1 (2.6)
Radiation only	7 (18.4)
No adjuvant therapy	7 (18.4)
Outcomes	
Median overall survival all patients months (IQR)	19.5 (2.25-28.75)
Alive at follow-up no. (%)	11 (28.9)
Median overall survival-months (IQR)	28 (23-37)
Deceased no. (%)	27 (71.1)
Median overall survival-months (IQR)	9 (2-25.5)

**Figure 2:**
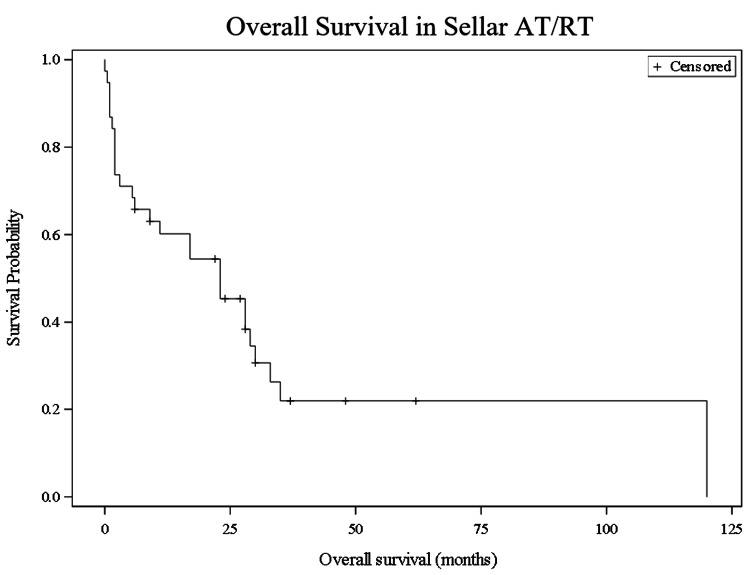
Kaplan-Meier survival curve of all patients. Overall survival was 65.8% at six months, 60.2% at one year, 45.4% at two years, and 21.9% at five years.

Immunohistochemistry demonstrated loss of INI1 expression in all but two patients. Presenting symptoms were reported in 15 cases and included headache with visual disturbance (10 cases, 67%) and visual changes alone (five cases, 33%). Duration of headache before presentation ranged from one week to three months, and duration of visual changes before presentation ranged from six days to five months. MRI findings were described in 27 patients. Tumor sizes ranged from 1.6 to 3.63 cm in their largest dimension and were most frequently reported as heterogeneously enhancing (n=18; 66.7%). Of those with imaging, 12 cases (44.4%) reported extension into the cavernous sinuses, and six cases reported cystic components (22.2%). Two cases presented with pituitary apoplexy with evidence of subarachnoid and intraventricular hemorrhage on imaging. Median OS for the total patient population was 19.5 months (IQR 2.25-28.75). OS was 65.8% at six months, 60.2% at one year, 45.4% at two years, and 21.9% at five years (Figure 2).

**Figure 3:**
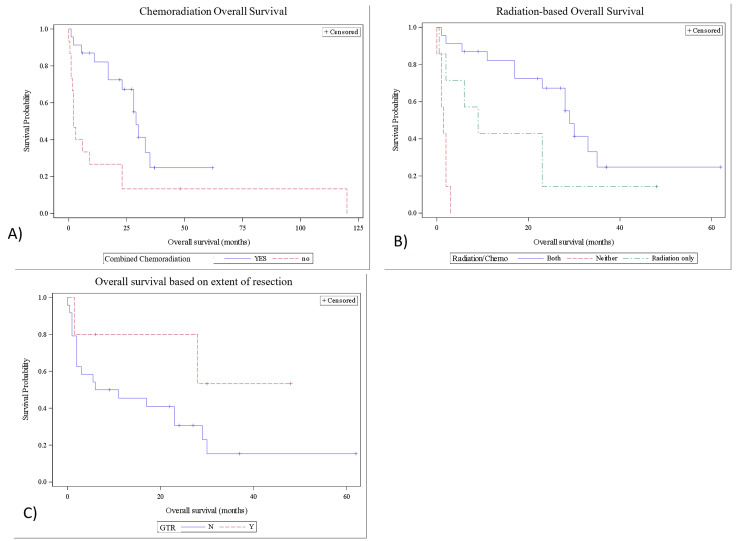
Kaplan-Meier survival curves for different treatment regimens. A) Combined chemoradiation vs. all other regimens. The log-rank test showed a significant difference in overall survival (p= 0.0052). B) Combined chemoradiation compared to radiation only and no adjuvant therapy. Chemotherapy only was not included in this survival analysis because only one patient received this therapy. The log-rank test showed a significant difference in overall survival (p= <0.0001). C) Kaplan-Meier survival curves for those who had gross total resection compared to those who did not. The log-rank test showed that there was no difference in overall survival (p=0.1474). GTR: gross total resection

With regard to treatment, gross total resection (GTR) was achieved in five cases (13%), subtotal resection (STR) in 24 cases (63%), and details were not reported in nine cases (24%). The GTR cohort had a 28-month (IQR=6-30) median OS compared to seven-and-a-half months in the STR cohort (IQR=2-23.25), but this difference was not statistically significant (p=0.15). Twenty-three patients (60.5%) received both adjuvant chemotherapy and radiation therapy (Tables 1, 2), with a median survival of 27 months (IQR=14-30). Overall survival in the chemoradiation group was 87.0% at six months, 82.1% at one year, 67.3% at two years, and 24.8% at five years. The remaining 15 patients’ treatment regimens included one patient with chemotherapy only (2.6%), seven patients with radiation therapy only (18.4%), and seven patients with neither (18.4%). The median overall survival for these 15 patients was two months (IQR=1.25-16). Overall survival for the patients who did not receive combined chemoradiation therapy was 33.3% at six months, 26.7% at one year, and 13.3% at two and five years. The difference in overall survival between these two groups was statistically significant (p=0.0052) (Figure 3).

The median OS of the radiation therapy alone group (n=7) was nine months (IQR=4-23), and the no adjuvant therapy group (n=7) had a median OS of 1.5 months (IQR= 1-2). Patients who received combined chemoradiation therapy had significantly increased overall survival compared to those who received only radiation therapy or neither (p=<0.0001) (Figure 3). In multivariate analysis, combined chemoradiation therapy (HR 0.137, 95% CI 0.046-0.409) and chemotherapy alone (HR 0.209, 95% CI 0.056-0.782) were statistically significant (Tables 3, 4). 

**Table 3:** Multivariate analysis with chemotherapy and radiation therapy as separate variables. GTR: gross total resection

Variable	Hazard ratio	95% Confidence Interval	p
Age	0.995	0.956-1.035	0.81
Chemo	0.209	0.056-0.782	0.02
Radiation	0.346	0.082-1.466	0.15
GTR	1.193	0.259-5.492	0.82

**Table 4:** Multivariate analysis with combined chemo-radiation therapy GTR: gross total resection

Variable	Hazard Ratio	95% Confidence Interval	P value
Age	0.992	0.951-1.035	0.720
Chemo-radiation	0.137	0.046-0.409	0.0004
GTR	1.373	0.297-6.352	0.685

**Figure 4:**
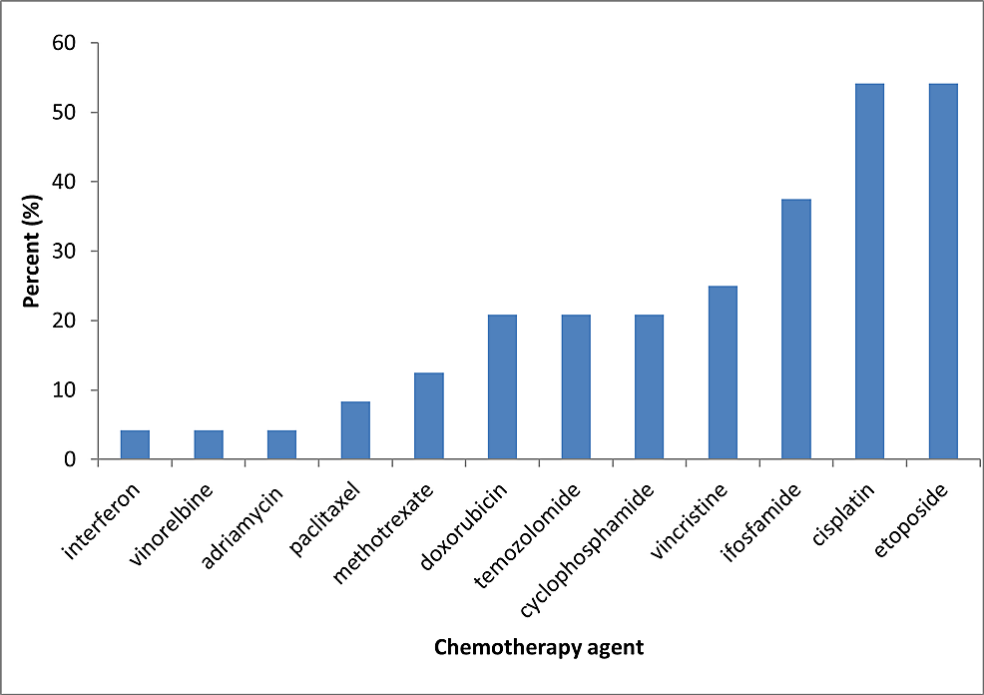
Frequency of different chemotherapy agents utilized.

Chemotherapy and radiotherapy regimens varied greatly between patients. Nineteen patients received multi-agent chemotherapy. The most common multi-agent chemotherapy regimen was ifosfamide, cisplatin, and etoposide (ICE) (8 patients). Nine patients (37.5%) received more than three chemotherapy agents, eight patients received three agent chemotherapy (33.3%), two received dual-agent chemotherapy (8.3%), two received single-agent chemotherapy (8.3%, temozolomide in both cases), and in three patients the chemotherapy regimen was not specified (12.5%). Two patients (8.3%) received high-dose chemotherapy followed by autologous stem cell transplantation. Fourteen patients received no chemotherapy. The most commonly included chemotherapy agents were etoposide (n=13; 54.2%), cisplatin (n=13; 54.2%), and ifosfamide (n=9; 37.5%) (Figure 4).

Intrathecal chemotherapy was used in three patients, with two receiving intrathecal methotrexate and our patient receiving intrathecal methotrexate, topotecan, etoposide, and thiotepa. The decision to use this combination of chemotherapy was based on previous studies demonstrating the efficacy of these agents on similar tumor types used in pediatric patients with AT/RT. In regards to radiation, 10 patients received focal radiation, seven patients received craniospinal radiation, seven patients had no radiation, five patients received stereotactic radiosurgery, and four patients received external beam therapy. Reported total radiation doses ranged from 20 Gy to 66 Gy.

Case example

**Figure 5:**
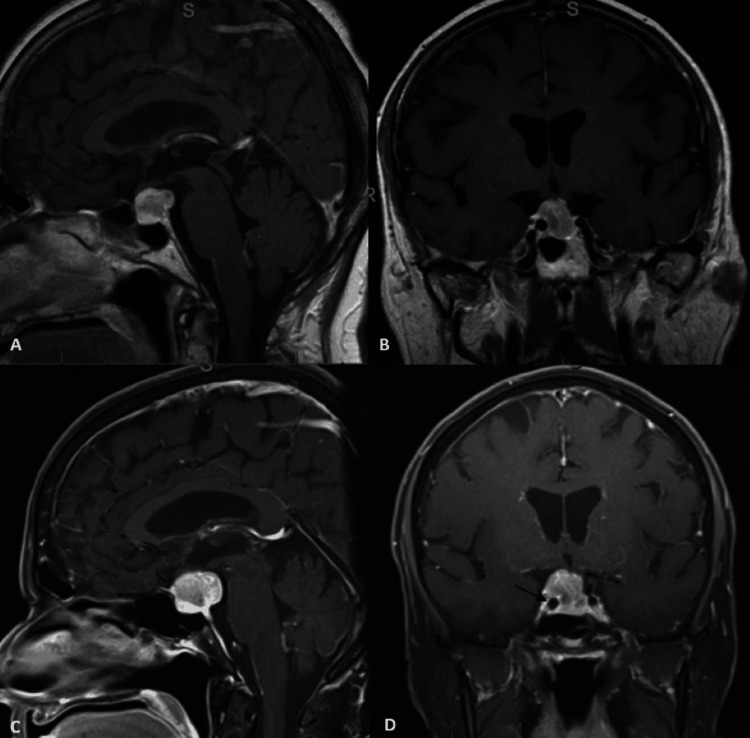
Top row: T1-weighted sagittal (A) and coronal (B) MRI images from one month prior revealed a 1.5 x 1.7 x 1.4 cm sellar/supprasellar lesion. Bottom row: T1-weighted sagittal (C) and coronal (D) MRI images at clinic presentation revealed a 1.8 x 2.2 x 1.7 cm heterogeneously enhancing sellar/suprasellar mass that extended through the suprasellar cistern encasing the right internal carotid artery (arrow).

Our patient was a 70-year-old woman with three months of increasingly severe headaches and four months of vision changes in the right eye, referred for management of a suspected pituitary adenoma. An ophthalmological evaluation six days before the presentation did not show papilledema or visual deficits. Visual fields were normal. Outpatient neurological examination revealed a dilated right pupil with mild ptosis and binocular vertical diplopia. The patient was hospitalized for further evaluation because of concerns about pituitary apoplexy or a non-pituitary adenoma-related process. Computed tomography angiography (CTA) and repeat MRI imaging revealed a 1.8 x 2.2 x 1.7 cm heterogeneously enhancing sellar/suprasellar mass that extended through the suprasellar cistern encasing the right internal carotid artery with no clear hemorrhage and demonstrated subtle progressive growth from an MRI scan one month prior (Figure 5).

The patient was placed on dexamethasone and underwent a subtotal endonasal transsphenoidal resection of the tumor the following day. Intraoperatively, despite many attempts to access lesional tissue through the gland, no frank abnormal lesional tissue was encountered. 

**Figure 6:**
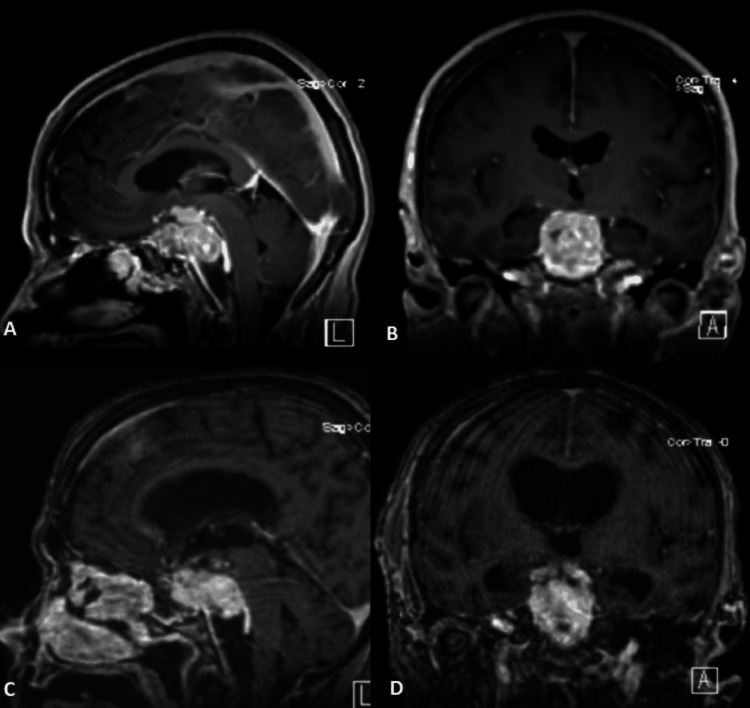
Top row: Post-endonasal approach sagittal (A) and coronal (B) T1 post-gadolinium MRI images revealed a subsequent increase of the sellar/suprasellar lesion (2.9 x 2.1 x 3.3 cm) with prepontine cisternal effacement, exhibiting aggressive growth with cavernous extension. Bottom row: Sagittal (C) and coronal (D) T1 post-gadolinium MRI images at the two-week follow-up after open craniotomy revealed continued enlargement of the residual lesion (2.6 x 3.3 x 4.0 cm) with optic nerve and brainstem compression as well as obstruction of the cerebral aqueduct with interval enlargement of the ventricular system.

The patient’s diplopia improved, and she was discharged on a postoperative day two with close follow-up with a plan for subsequent craniotomy. Additional workups, including systemic imaging and diagnostic lumbar puncture, were unremarkable. A two-week post-operative MRI scan revealed a substantial interval increase of the sellar/suprasellar lesion (2.9 x 2.1 x 3.3 cm) with prepontine cisternal effacement and extension into the cavernous sinus (Figure 6).

**Figure 7:**
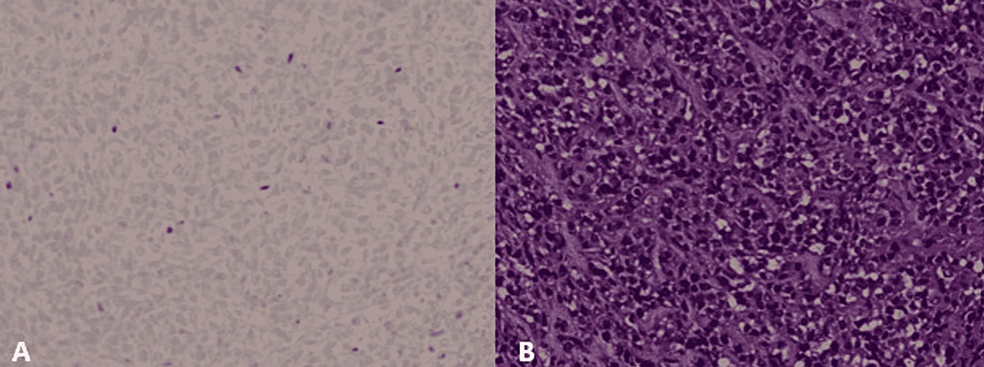
Histopathology of the lesion. Image A showing INI-1 negativity. Image B shows dense, diffuse proliferation of cells with vesicular nuclei and prominent nucleoli

The patient wished for additional recovery time, and two weeks after the initial surgery, a right frontotemporal approach was utilized to achieve a subtotal resection of the lesion. Pathology revealed a SMARCB/INI-1 deficient atypical teratoma/rhabdoid tumor (Figure 7).

Two weeks post-operatively, altered mental status and electrolyte imbalance secondary to diabetes insipidus prompted re-admission. An MRI at this time revealed enlargement of the residual lesion (2.6 x 3.3 x 4.4 cm) with optic nerve and brainstem compression, perilesional blood products, and progressive periventricular T2 hyperintensities (Figure 6). The patient was admitted, and urgent inpatient radiation was initiated. The patient ultimately received 30 Gy in 10 fractions over 13 elapsed days, with concurrent cisplatin administered on day seven of radiation. 

Unfortunately, the patient’s neurological status continued to deteriorate with progressive headaches, right eye ophthalmoplegia, and decreasing visual acuity. On workup for acute mental status changes during the radiation therapy, serial electroencephalograms (EEG) revealed right-sided intermittent epileptiform activity and lateralized periodic discharges requiring continued hospitalization for anti-epileptic treatment.

**Figure 8:**
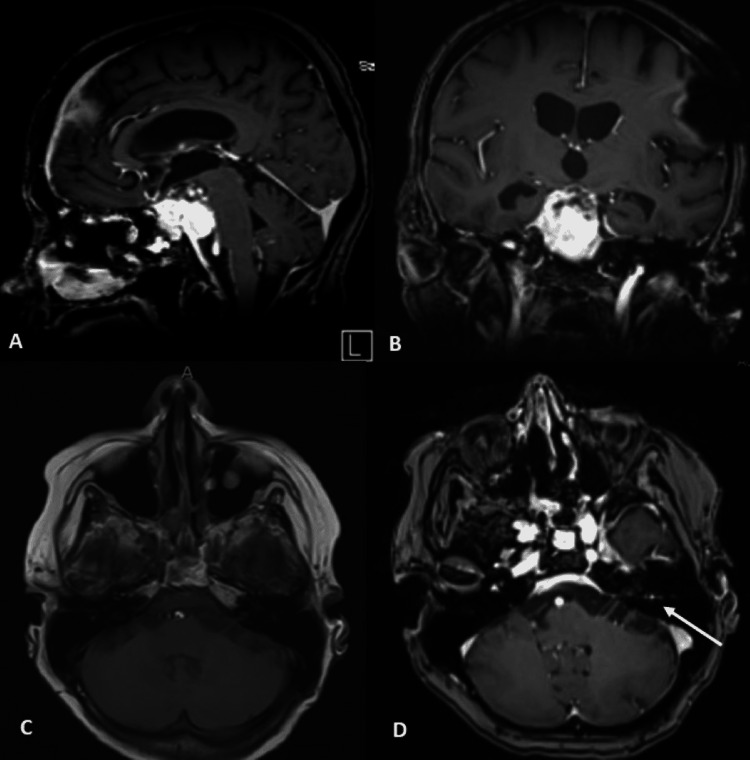
Top row: T1 post-contrast sagittal (A) and coronal (B) MRI images 2 months after initiation of the radiation therapy showed control and a slight decrease in solid tumor growth. Bottom row: T1 pre-contrast (C) and post-contrast (D) axial MRI. New enhancement was seen along the left facial nerve two months after initiation of radiation therapy concerning for leptomeningeal disease.

Six weeks following the craniotomy, the patient developed obstructive hydrocephalus requiring a right frontal ventriculoperitoneal shunt (VPS). Repeat MRI imaging two months after initiation of the radiation therapy showed a slight decrease in the solid tumor mass. However, there was a new enhancement along the left facial nerve concerning leptomeningeal metastases, corroborated by subsequent positive CSF cytology (Figure 8). 

In light of these findings, treatment with intravenous carboplatin and etoposide along with multi-agent intraventricular chemotherapy (alternating cycles of etoposide plus topotecan and methotrexate plus thiotepa) was initiated. The patient’s ventriculoperitoneal shunt was converted to an Ommaya reservoir/Certas programmable ventriculoperitoneal shunt to facilitate effective intraventricular chemotherapy administration. Five months after the initial surgery, the patient developed a left subdural hematoma, likely secondary to dural-based metastases, requiring emergent burr-hole evacuation. MRI showed further tumor progression, with multiple dural-based nodular enhancing lesions in the left parietal superolateral convexity indicative of dural metastases. At this point, she had received three cycles of intraventricular and one cycle of intravenous chemotherapy. Cerebrospinal fluid (CSF) cytology on the last sampling was still positive for malignant cells. Post-operatively, the patient remained disoriented, with a deteriorating neurological status. Nine days post-operatively, the family elected for hospice and comfort care. The patient passed comfortably approximately five-and-half months after initial evaluation, nine-and-half months after visual acuity changes, and eight-and-half months after headache presentation.

Discussion

Our search uncovered fewer than 50 reported cases of female predominant sellar AT/RT. Most reported cases provided SMARCB1/INI1 expression, adjuvant treatments, and radiographic findings. While adjuvant treatment types varied greatly between individuals, those who underwent adjuvant chemotherapy and radiation therapy had significantly longer survival times compared to those who did not.

Similar to Chan et al., cases of sellar AT/RT reported in the literature showed a strong female predominance (n=36; 94.7%), in contrast to pediatric AT/RTs and adult non-sellar AT/RTs, for which there is a less striking 1.3-2.0:1 male predominance [2,3,9-11,12,29]. There was a wide age range (20 to 70 years old), with our patient being the oldest reported patient with sellar AT/RT. 

Imaging findings in sellar AT/RT are not pathognomonic, and radiographic features often overlap with those of pituitary macroadenomas [43-48]. AT/RTs are usually larger than pituitary adenomas at presentation, with those reported in the literature ranging from 1.6 cm to 3.63 cm. Similar to pituitary adenomas, they are isointense on T1-weighted images with heterogeneous contrasted enhancement. Heterogeneous enhancement on MRI was reported in 66.7% of sellar AT/RT cases in our study, comparable to 70% of all adult AT/RTs reported by Kanoto et al. 44.4% of cases in this review described cavernous sinus invasion (n=12), and 22.2 % described cystic portions of the tumor, both features that are also seen in aggressive pituitary macroadenomas [43-48]. Lev et al. reported that, rather than imaging findings, the most defining feature before histological confirmation is the length of symptoms at presentation [22]. Due to the aggressive nature of these tumors, sellar AT/RTs had a rapid progression of headaches and vision changes before the presentation. The reported duration of headaches ranged from 10 days to two months, and visual changes from six days to two months. Our systematic review found a range of one week to three months for the duration of headaches before the presentation and a range of six days to five months of visual changes before the presentation. This contrasts with pituitary macroadenomas, where the pre-diagnosis duration of headaches and visual changes are 12 months and 3-5 months, respectively [49-51].

All but two cases of sellar AT/RT reported the loss of SMARCB1/INI1 expression on immunohistochemistry. Multiple mutations have been reported, all involving the SMARCB1 gene on chromosome 22q11 [29,32,34,35,43]. These include loss of heterozygosity, stop codon point mutations, frameshift mutations, frameshift deletions, and homozygous deletions encompassing SMARCB1 [29,32,34,39]. The loci of mutations are in exons two and four of SMARCB1 for adult AT/RT but in exons five and nine in childhood AT/RT [35]. In addition to mutations in the SMARCB1 gene, three different methylation patterns of CG sites have been described in childhood AT/RT, including AT/RT-TYR, AT/RT-SHH, and AT/RT-MYC [32]. AT/RT-TYR and AT/RT-SHH are characterized by increased methylation of CG sites, while AT/RT-MYC is characterized by decreased methylation. Johann et al. found that sellar ATRT has statistically similar methylation patterns to AT/RT-MYC [32]. Molecular testing of our patient’s tumor did not demonstrate microsatellite instability, and the tumor mutational burden was low. SMARCB1 had indeterminate results with next-generation sequencing, likely related to low coverage of some or all exons of the gene. 

Histologically, sellar AT/RTs show dense, diffuse proliferation of small- to medium-sized cells with vesicular nuclei and prominent nucleoli [29, 43]. Cells can be arranged in sheets or lobules and are variable in cytoplasmic features, including varying uptake of eosin [43]. Scattered rhabdoid cells and staghorn vasculature is seen in many cases, with Nakata et al. reporting all six samples demonstrated the latter [29]. A high Ki-67 labeling index is seen [43]. Immunohistochemistry demonstrates variability in the expression of EMA, CAM5.2, and GFAP [29, 43]. However, Nakata et al.’s study of six case samples was negative for INI1 and STAT6 and positive for the expression of vimentin and α-smooth muscle actin (α-SMA) [29]. On immunohistochemistry, our patient had a Ki-67 of approximately 60%, was INI1 and GFAP negative, and CAM5.2, EMA, and α-SMA positive.

Adjuvant treatment regimens for adult sellar AT/RT varied greatly in the reported cases, with 63.2% of cases receiving chemotherapy, 78.9% of cases receiving radiation therapy, 60.5% receiving combined chemoradiation therapy, and 18.4% receiving no adjuvant therapies. The lack of a standardized chemotherapy protocol for adult AT/RT is evident, with various regimens detailed in our review. Most chemotherapy regimens are based on protocols for childhood AT/RT; the most common protocols are described in Table 5 [7,12,52-58]. Broadly, these protocols involve maximal safe surgical resection before a multi-agent induction and maintenance chemotherapy regimen with adjuvant radiotherapy. 

**Table 5:** Most common chemotherapy regimens utilized in AT/RT treatment [6, 52-57].

Protocol	Regimen
Children’s Oncology Group (COG) 99703 [52]	Following biopsy/resection, three identical cycles of induction chemotherapy (vincristine, cyclophosphamide, etoposide and cisplatin) administered every 21-28 days. Patients without tumor progression then received three consolidation cycles of marrow-ablative chemotherapy (thiotepa and carboplatin) followed by autologous hematopoietic cell rescue (AuHCR).
Pediatric Brain Tumor Consortium (PBTC)- 001 [53]	Following resection of tumor, 20 weeks of induction chemotherapy consisting of vincristine, cisplatin, cyclophosphamide, oral etoposide and intrathecal mafosfamide. Patients with no metastatic disease at diagnosis (M0) proceed to local conformal irradiation therapy. Following local irradiation, an additional 12 weeks of adjuvant vincristine, cyclophosphamide, and oral etoposide chemotherapy are given.
Children’s Cancer Group (CCG 9921) [54]	Following resection of tumor, induction therapy (five cycles, each three weeks in duration) consisting of: Regimen A: Vincristine, Cisplatin, Cyclophosphamide, and etoposide. Regimen B: Vincristine, carboplatin, ifosfamide Granulocyte colony-stimulating factor (G-CSF) administered within 24 hours of completion of chemotherapy. Maintenance chemotherapy (eight cycles, each cycle 49 days), Vincristine, etoposide, carboplastin, cyclophosphamide. Those with persistent residual disease after induction or metastatic disease at diagnosis receive radiation therapy when they reach 36 months or at completion of eight cycles of maintenance chemotherapy (whichever comes first).
Headstart III [55]	Following maximal surgical resection, five induction cycles of Vincristine, cisplatin, cyclophosphamide, etoposide, and high dose methotrexate in cycles 1, 3, and 5; Vincristine, cyclophosphamide, oral etoposide, and temozolomide in cycles 2 and 4. If residual tumor at the completion of induction, second look surgery followed by consolidation with myeloablative chemotherapy (thiotepa, carboplatin, and etoposide) with autologous hematopoietic cell rescue (Au-HCR). In children without tumor progression, induction was followed by consolidation with myeloablative chemotherapy (thiotepa, carboplatin, and etoposide) with Au-HCR.
Intergroup Rhabdomyosarcoma Study III (IRS III)-regimen 36 [56]	Following maximal surgical resection, weekly vincristine during radiation, actinomycin-D, doxorubicin, and triple intrathecal chemotherapy with hydrocortisone, methotrexate, and cytosine arabinoside.
Medical University of Vienna AT/RT protocol [6]	Following maximal safe resection, three nine-week courses of doxorubicin, cyclophosphamide, vincristine, ifosfamide, cisplatin, etoposide, and methotrexate, augmented by intrathecal therapy and high dose chemotherapy (HDCT) carboplatin, etoposide, and thiotepa with AuHCR Local radiotherapy completed six weeks after HDCT
St. Jude’s AT/RT Protocol [57]	Following maximal surgical resection, four cycles of cisplatin with high dose cyclophosphamide and vincristine followed by Au-HCR

In our analysis, patients who underwent combination chemotherapy and radiation therapy had the longest survival. This survival benefit was still significant when compared to radiation therapy alone and no adjuvant treatment, consistent with prior reports for all adult AT/RT [12]. The advantage of chemotherapy after surgery further validates similar results in pediatric AT/RT management [2-11].

In pediatric patients, gross total resection, compared to subtotal resection, provides a statistically higher OS [2,7]. Although median survivals in the adult sellar AT/RT population differed greatly between GTR and STR (median OS 28 vs. 7.5 months), the rarity of the disease prevents statistical significance (p=0.15). This finding is consistent with Chan et al., who found no significant difference when comparing gross total resection to biopsy or subtotal resection in all adult AT/RT cases [12]. In pediatric AT/RT, Lau et al. report a significant increase in overall survival when comparing combined surgery and radiation to surgery alone, radiation alone, and no treatment [11]. OS benefit from radiation has been confirmed repeatedly in the pediatric population [5,7,8,10]. Our analysis also suggests increased overall survival in adult sellar AT/RT with postoperative adjuvant radiation therapy.

In addition to high-dose chemotherapy, autologous stem cell replacement and intrathecal chemotherapy are also utilized in childhood AT/RT with significantly increased OS benefits [7]. In an analysis of overall survival in patients who received chemotherapy based on the Children’s Oncology Group (COG) 99703 protocol, Children’s Cancer Group (CCG) 9921 protocol, and Intergroup Rhabdomyosarcoma study III protocol, Hilden et al. found 14 of 42 pediatric patients to be long-term survivors (alive with no evidence of disease at the time of publication) with a median event-free survival of 42 months (range, 11 to 90 months) from diagnosis [2,52,54,55]. 71.4% of long-term survivors had a gross total resection, 50% received radiotherapy, 42.9% received intrathecal (IT) chemotherapy and 28.6% received high dose chemotherapy (HDCT) with autologous hematopoietic cell rescue (AuHCR). The two patients that received HDCT with AuHCR in our analysis showed long-term survival, 120 months (death unrelated to AT/RT) and 30 months (alive). Both of these survivals were greater than the median overall survival of 19.5 months. Additionally, only three cases reported the use of IT chemotherapy, including our patient, surviving 28 months (deceased), 33 months (deceased), and five-and-half months (deceased). The two long-term survivors receiving IT chemotherapy were younger than our patient (31 and 26 years old vs. 70 years old); our patient died a month after initiation of IT chemotherapy and 15 days after initiation of systemic intravenous chemotherapy. Our patient began concurrent cisplatin on fraction 7 of 10 of radiotherapy, with IT chemotherapy and systemic chemotherapy starting four months and four-and-half months, respectively, after initial diagnosis. The significance of intrathecal versus intravenous chemotherapy cannot be extrapolated from the case reports but should be further explored especially given the precedent set by treatment in pediatric AT/RT patients.

Limitations

Our study is limited by the small cohort available in the literature, given the rarity of adult sellar AT/RT. Each publication differed greatly in the quality of patient characteristics, MRI findings, treatment regimens, and reported outcomes. Therefore, it is difficult to assess for any confounding factors that may be present within this analysis. Unfortunately, the data that was available to review in the published studies did not report key covariates, such as Karnofsky Performance Score, that have been shown to be associated with both aggressive tumor-directed therapy and survival.

## Conclusions

Adult sellar atypical teratoid/rhabdoid tumor is a rare malignancy that carries a poor prognosis. Current data does not statistically support GTR over STR; however, there is potential that this finding could be due to the small sample size. The best overall survival was achieved via adjuvant chemotherapy and radiation. Early consideration of neuro-oncology and radiation oncology referral and management is likely beneficial in this patient population. Intrathecal chemotherapy is a treatment modality that needs further study given the limited options and current dismal prognosis of adult sellar AT/RT.

## References

[1] Nesvick CL, Nageswara Rao AA, Raghunathan A, Biegel JA, Daniels DJ (2019). Case-based review: atypical teratoid/rhabdoid tumor. Neurooncol Pract.

[2] Hilden JM, Meerbaum S, Burger P (2004). Central nervous system atypical teratoid/rhabdoid tumor: results of therapy in children enrolled in a registry. J Clin Oncol.

[3] Athale UH, Duckworth J, Odame I, Barr R (2009). Childhood atypical teratoid rhabdoid tumor of the central nervous system: a meta-analysis of observational studies. J Pediatr Hematol Oncol.

[4] Lafay-Cousin L, Hawkins C, Carret AS (2012). Central nervous system atypical teratoid rhabdoid tumours: the Canadian Paediatric Brain Tumour Consortium experience. Eur J Cancer.

[5] Chen YW, Wong TT, Ho DM, Huang PI, Chang KP, Shiau CY, Yen SH (2006). Impact of radiotherapy for pediatric CNS atypical teratoid/rhabdoid tumor (single institute experience). Int J Radiat Oncol Biol Phys.

[6] Slavc I, Chocholous M, Leiss U (2014). Atypical teratoid rhabdoid tumor: improved long-term survival with an intensive multimodal therapy and delayed radiotherapy. The Medical University of Vienna Experience 1992-2012. Cancer Med.

[7] Schrey D, Carceller Lechón F, Malietzis G (2016). Multimodal therapy in children and adolescents with newly diagnosed atypical teratoid rhabdoid tumor: individual pooled data analysis and review of the literature. J Neurooncol.

[8] Ostrom QT, Chen Y, M de Blank P (2014). The descriptive epidemiology of atypical teratoid/rhabdoid tumors in the United States, 2001-2010. Neuro Oncol.

[9] Quinn TJ, Almahariq MF, Siddiqui ZA (2019). Trimodality therapy for atypical teratoid/rhabdoid tumor is associated with improved overall survival: A surveillance, epidemiology, and end results analysis. Pediatr Blood Cancer.

[10] Buscariollo DL, Park HS, Roberts KB, Yu JB (2012). Survival outcomes in atypical teratoid rhabdoid tumor for patients undergoing radiotherapy in a surveillance, epidemiology, and end results analysis. Cancer.

[11] Lau CS, Mahendraraj K, Chamberlain RS (2015). Atypical teratoid rhabdoid tumors: a population-based clinical outcomes study involving 174 patients from the Surveillance, Epidemiology, and End Results database (1973-2010). Cancer Manag Res.

[12] Chan V, Marro A, Findlay JM, Schmitt LM, Das S (2018). A systematic review of atypical teratoid rhabdoid tumor in adults. Front Oncol.

[13] Kuge A, Kayama T, Tsuchiya D, Kawakami K, Saito S, Nakazato Y, Suzuki H (2000). [Suprasellar primary malignant rhabdoid tumor in an adult: a case report]. No Shinkei Geka.

[14] Raisanen J, Biegel JA, Hatanpaa KJ, Judkins A, White CL, Perry A (2005). Chromosome 22q deletions in atypical teratoid/rhabdoid tumors in adults. Brain Pathol.

[15] Arita K, Sugiyama K, Sano T, Oka H (2008). Atypical teratoid/rhabdoid tumour in sella turcica in an adult. Acta Neurochir (Wien).

[16] Las Heras F, Pritzker KP (2010). Adult variant of atypical teratoid/rhabdoid tumor: immunohistochemical and ultrastructural confirmation of a rare tumor in the sella tursica. Pathol Res Pract.

[17] Schneiderhan TM, Beseoglu K, Bergmann M (2011). Sellar atypical teratoid/rhabdoid tumours in adults. Neuropathol Appl Neurobiol.

[18] Chou SQH, Lo SSM, Wong HN (2013). Atypical teratoid/rhabdoid tumour in the sella turcica of a female adult. Hong Kong J Radiol.

[19] Moretti C, Lupoi D, Spasaro F (2013). Sella turcica atypical teratoid/rhabdoid tumor complicated with lung metastasis in an adult female. Clin Med Insights Case Rep.

[20] Park HG, Yoon JH, Kim SH, Cho KH, Park HJ, Kim SH, Kim EH (2014). Adult-onset sellar and suprasellar atypical teratoid rhabdoid tumor treated with a multimodal approach: a case report. Brain Tumor Res Treat.

[21] Shitara S, Akiyama Y (2014). Atypical teratoid/rhabdoid tumor in sellar turcica in an adult: A case report and review of the literature. Surg Neurol Int.

[22] Lev I, Fan X, Yu R (2015). Sellar atypical teratoid/rhabdoid tumor: Any preoperative diagnostic clues?. AACE Clin Case Reports.

[23] Biswas S, Wood M, Joshi A (2015). Exome sequencing of an adult pituitary atypical teratoid rhabdoid tumor. Front Oncol.

[24] Regan JM, Tehrani M, Rodriguez FJ, Watson JC: Adult AT/ RT (2015). Predilection for the pituitary?. J Neuro Neuros.

[25] Nobusawa S, Nakata S, Hirato J (2016). Atypical teratoid/rhabdoid tumor in the sella turcica of an elderly female with a distinct vascular pattern and genetic alterations. Virchows Arch.

[26] Almalki MH, Alrogi A, Al-Rabie A, Al-Dandan S, Altwairgi A, Orz Y (2017). Atypical teratoid/rhabdoid tumor of the sellar region in an adult with long survival: Case report and review of the literature. J Clin Med Res.

[27] Larrán-Escandón L, Mateo-Gavira I, Vilchez-López FJ, Gómez Cárdenas E, Aguilar Diosdado M (2016). Pituitary apoplexy as presentation of atypical teratoid/rhabdoid tumor in an adult. Endocrinol y Nutr (English Ed.

[28] Elsayad K, Kriz J, Samhouri L, Haverkamp U, Straeter R, Stummer W, Eich HT (2016). Long-term survival following additive radiotherapy in patients with atypical teratoid rhabdoid tumors. Strahlenther Onkol.

[29] Nakata S, Nobusawa S, Hirose T (2017). Sellar atypical teratoid/rhabdoid tumor (AT/RT): A clinicopathologically and genetically distinct variant of AT/RT. Am J Surg Pathol.

[30] Dardis C, Yeo J, Milton K (2017). Atypical teratoid rhabdoid tumor: Two case reports and an analysis of adult cases with implications for pathophysiology and treatment. Front Neurol.

[31] Pratt D, Mehta GU, Wang HW, Chittiboina P, Quezado M (2017). A 47-year old female with a destructive sellar mass. Brain Pathol.

[32] Johann PD, Bens S, Oyen F (2018). Sellar region atypical teratoid/rhabdoid tumors (ATRT) in adults display DNA methylation profiles of the ATRT-MYC subgroup. Am J Surg Pathol.

[33] Nishikawa A, Ogiwara T, Nagm A (2018). Atypical teratoid/rhabdoid tumor of the sellar region in adult women: Is it a sex-related disease?. J Clin Neurosci.

[34] Paolini MA, Kipp BR, Sukov WR (2018). Sellar region atypical teratoid/rhabdoid tumors in adults: Clinicopathological characterization of five cases and review of the literature. J Neuropathol Exp Neurol.

[35] Barresi V, Lionti S, Raso A, Esposito F, Cannavò S, Angileri FF (2018). Pituitary atypical teratoid rhabdoid tumor in a patient with prolactinoma: A unique description. Neuropathology.

[36] Su HY, Su YF (2018). A 37-year-old woman with progressive right side ptosis for one month. Brain Pathol.

[37] Barsky D (2018). Sellar atypical teratoid rhabdoid tumor (Atrt) in an adult: A case report and review of the literature. Biomed J Sci Tech Res.

[38] Asmaro K, Arshad M, Massie L, Griffith B, Lee I (2019). Sellar atypical teratoid/rhabdoid tumor presenting with subarachnoid and intraventricular hemorrhage. World Neurosurg.

[39] Voisin MR, Ovenden C, Tsang DS (2019). Atypical teratoid/rhabdoid sellar tumor in an adult with a familial history of a germline SMARCB1 mutation: Case report and review of the literature. World Neurosurg.

[40] Siddiqui M, Thoms D, Samples D, Caron J (2019). Atypical teratoid/rhabdoid tumor presenting with subarachnoid and intraventricular hemorrhage. Surg Neurol Int.

[41] Lawler K and Robertson T (2019). A rare case of sellar region atypical teratoid/rhabdoid tumour in an adult female. Pathology.

[42] Bokhari R, Bafaqeeh M, Al-Obaysi S, Al-Aman A, Alshakweer W (2020). Atypical teratoid/rhabdoid tumor of the sellar region: A case report and review of the literature. J Neuro Res.

[43] Roncaroli F, Villa C, Chatterjee D (2019). Rare primary non-neuroendocrine tumours of the sella. Diagnostic Histopathol.

[44] Rennert J, Doerfler A (2007). Imaging of sellar and parasellar lesions. Clin Neurol Neurosurg.

[45] Tosaka M, Sato N, Hirato J (2007). Assessment of hemorrhage in pituitary macroadenoma by T2*-weighted gradient-echo MR imaging. AJNR Am J Neuroradiol.

[46] Knosp E, Steiner E, Kitz K, Matula C (1993). Pituitary adenomas with invasion of the cavernous sinus space: a magnetic resonance imaging classification compared with surgical findings. Neurosurgery.

[47] Micko AS, Wöhrer A, Wolfsberger S, Knosp E (2015). Invasion of the cavernous sinus space in pituitary adenomas: endoscopic verification and its correlation with an MRI-based classification. J Neurosurg.

[48] Kanoto M, Toyoguchi Y, Hosoya T, Kuchiki M, Sugai Y (2015). Radiological image features of the atypical teratoid/rhabdoid tumor in adults: a systematic review. Clin Neuroradiol.

[49] Gondim JA, de Almeida JP, de Albuquerque LA, Schops M, Gomes E, Ferraz T (2009). Headache associated with pituitary tumors. J Headache Pain.

[50] Jahangiri A, Lamborn KR, Blevins L, Kunwar S, Aghi MK (2012). Factors associated with delay to pituitary adenoma diagnosis in patients with visual loss. J Neurosurg.

[51] Gnanalingham KK, Bhattacharjee S, Pennington R (2005). The time course of visual field recovery following transphenoidal surgery for pituitary adenomas: predictive factors for a good outcome. Journal of Neurology, Neurosurgery & Psychiatry.

[52] Cohen BH, Geyer JR, Miller DC (2015). Pilot study of intensive chemotherapy with peripheral hematopoietic cell support for children less than 3 years of age with malignant brain tumors, the CCG-99703 phase I/II study. A report from the Children's Oncology Group. Pediatr Neurol.

[53] Blaney SM, Kocak M, Gajjar A (2012). Pilot study of systemic and intrathecal mafosfamide followed by conformal radiation for infants with intracranial central nervous system tumors: a pediatric brain tumor consortium study (PBTC-001). J Neurooncol.

[54] Geyer JR, Sposto R, Jennings M (2005). Multiagent chemotherapy and deferred radiotherapy in infants with malignant brain tumors: a report from the Children's Cancer Group. J Clin Oncol.

[55] Zaky W, Dhall G, Ji L (2014). Intensive induction chemotherapy followed by myeloablative chemotherapy with autologous hematopoietic progenitor cell rescue for young children newly-diagnosed with central nervous system atypical teratoid/rhabdoid tumors: the Head Start III experience. Pediatr Blood Cancer.

[56] Crist W, Gehan EA, Ragab AH (1995). The Third Intergroup Rhabdomyosarcoma Study. J Clin Oncol.

[57] Tekautz TM, Fuller CE, Blaney S (2005). Atypical teratoid/rhabdoid tumors (ATRT): improved survival in children 3 years of age and older with radiation therapy and high-dose alkylator-based chemotherapy. J Clin Oncol.

[58] Olson TA, Bayar E, Kosnik E, Hamoudi AB, Klopfenstein KJ, Pieters RS, Ruymann FB (1995). Successful treatment of disseminated central nervous system malignant rhabdoid tumor. J Pediatr Hematol Oncol.

